# Tumor Necrosis Factor Alpha Contributes to Inflammatory Pathology in the Placenta during Brucella abortus Infection

**DOI:** 10.1128/iai.00013-22

**Published:** 2022-03-17

**Authors:** April Y. Tsai, Mariana X. Byndloss, Núbia Seyffert, Maria G. Winter, Briana M. Young, Renée M. Tsolis

**Affiliations:** a Department of Medical Microbiology and Immunology, School of Medicine, University of California at Davis, Davis, California, USA; University of California—San Diego School of Medicine

**Keywords:** *Brucella*, inflammation, placental immunology, zoonotic infections

## Abstract

Research on Brucella pathogenesis has focused primarily on its ability to cause persistent intracellular infection of the mononuclear phagocyte system. At these sites, Brucella abortus evades innate immunity, which results in low-level inflammation and chronic infection of phagocytes. In contrast, the host response in the placenta during infection is characterized by severe inflammation and extensive extracellular replication of B. abortus. Despite the importance of reproductive disease caused by Brucella infection, our knowledge of the mechanisms involved in placental inflammation and abortion is limited. To understand the immune responses specifically driving placental pathology, we modeled placental B. abortus infection in pregnant mice. B. abortus infection caused an increase in the production of tumor necrosis factor alpha (TNF-α), specifically in the placenta. We found that placental expression levels of *Tnfa* and circulating TNF-α were dependent on the induction of endoplasmic reticulum stress and the B. abortus type IV secretion system (T4SS) effector protein VceC. Blockade of TNF-α reduced placental inflammation and improved fetal viability in mice. This work sheds light on a tissue-specific response of the placenta to B. abortus infection that may be important for bacterial transmission via abortion in the natural host species.

## INTRODUCTION

Human brucellosis, caused most commonly by Brucella abortus and B. melitensis, is considered one of the most important zoonotic diseases worldwide and results in significant morbidity and economic impacts (1). A key feature of human brucellosis is persistent bacterial colonization of the mononuclear phagocyte system. In its natural bovine host, B. abortus is transmitted by contact between susceptible individuals and the contaminated fetus, fetal membranes, and uterine secretions from an ill animal. Zoonotic transmission between bovines and humans can occur through direct contact with infected animals or from the consumption of contaminated dairy products (2, 3) since bacteria can be shed in the milk.

Since using the natural host as a model to study animal brucellosis presents significant challenges, including biosafety concerns, high cost, and the requirement of valid biocontainment facilities, mouse models can be utilized to study how Brucella spp. persist in systemic organs and interact with placental tissues in pregnant animals (4, 5). In the murine persistence model, in which the pathogens primarily localize within phagocytic cells from tissues such as the liver, spleen, and lymph nodes, the infection elicits the formation of granuloma-like structures and low-level inflammation (2). However, in the pregnant mouse model, in which B. abortus targets trophoblast giant cells during infection, the bacteria replicate to very high levels in the placenta and cause necrotizing inflammation of the placenta (2, 6, 7). Studies have linked this inflammation to abortion since the blockade of two proinflammatory cytokines, interferon gamma and regulated upon activation normal T-cell expressed and secreted (RANTES), rescued fetal loss in Brucella-infected pregnant mice (4, 8). Despite the importance of reproductive disease caused by B. abortus for agriculture and transmission to humans, very little is known about the immune responses elicited in the infected placenta as well as how the placental response differs from that described for other infected tissues. Interestingly, our previous study discovered a remarkable phenotypic difference in tissue pathology when comparing the infected placenta and the infected spleen. While no histological evidence of cell death was observed in splenic tissue, moderate to severe cell death was observed in the placenta (9). These results prompted us to further investigate the distinctive immune responses contributing to severe placental inflammation during B. abortus infection.

## RESULTS

### B. abortus induces the expression of *Tnfa* in the placenta but not the spleen.

**FIG 1:**
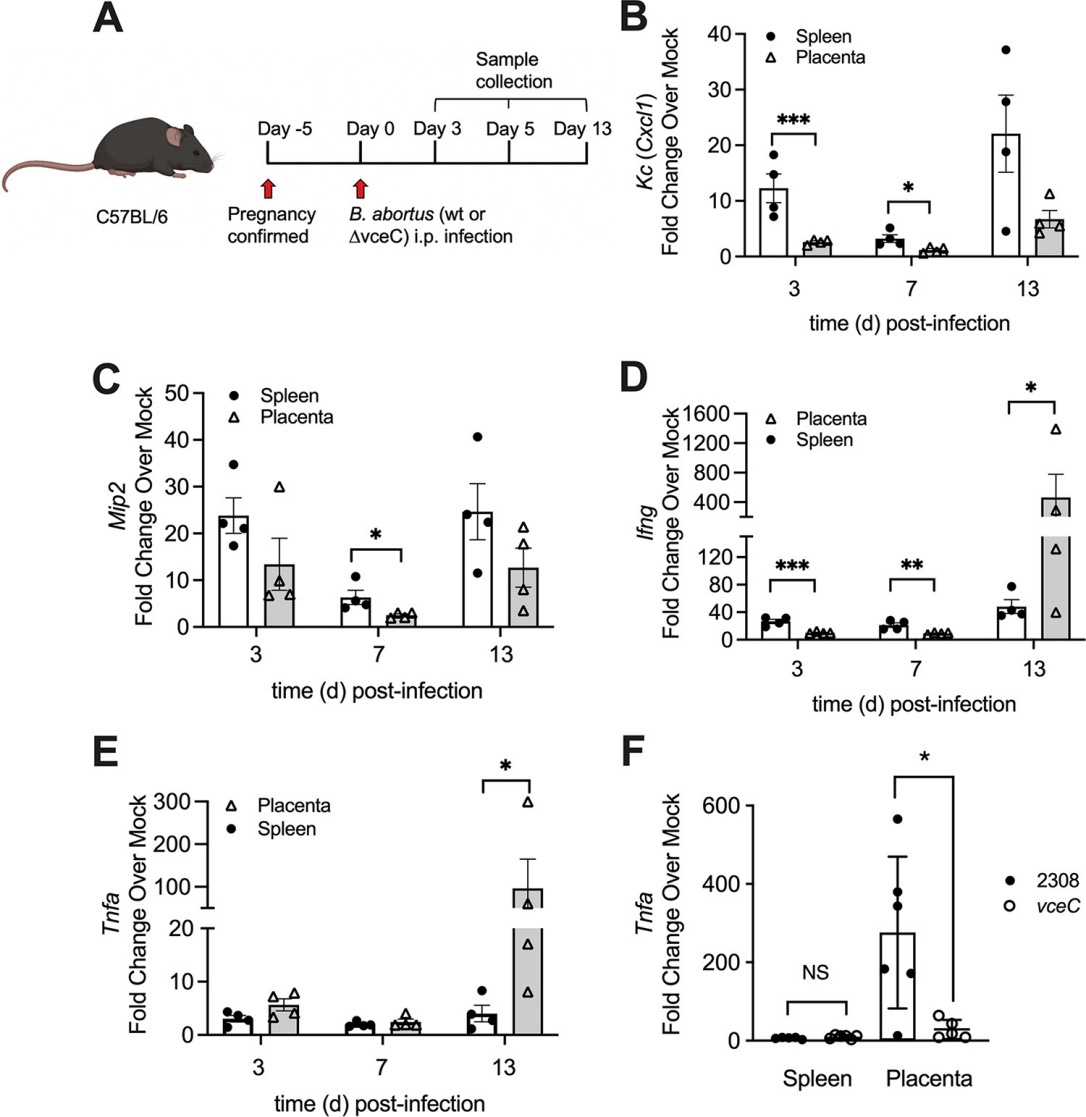
B. abortus induced TNF-α expression significantly in the placenta. (A) Schematic representation of the pregnant mouse model. (B to E) Real-time RT-PCR analysis of gene expression in spleens and placentas from pregnant mice infected with wild-type (wt) B. abortus for 3, 7, or 13 days (*n* = 4). Values represent means ± standard errors of the means (SEM). (F) Real-time RT-PCR analysis of *Tnfa* gene expression in placentas or spleens from pregnant mice infected for 13 days with B. abortus 2308 or its isogenic *vceC* mutant strain MDJ32 (*n* = 5). Values represent means ± SEM. *, *P* < 0.05; **, *P* < 0.01; ***, *P* < 0.001; NS, not significant (using Student’s *t* test on log-transformed data).

During the acute stage of Brucella infection in mice, the production of Th1 cytokines is upregulated by the host immune system (4, 10). However, these proinflammatory cytokines have also been shown to induce abortion in both infectious and noninfectious contexts and thus may be detrimental to pregnancy (11, 12). Our recent studies discovered differences in pathology between splenic and placental tissues during Brucella infection of pregnant mice, in which splenic tissue exhibits the formation of microgranulomas, whereas infected placental tissue is characterized by severe inflammation with neutrophil influx and necrosis (9). Therefore, based on the fact that Th1 cytokines are crucial for controlling Brucella infection but have also been implicated in noninfectious abortion, we aimed to further investigate the expression of proinflammatory genes in the infected spleen and placenta during infection of pregnant mice. To this end, we infected pregnant C57BL/6J mice with B. abortus via the intraperitoneal (i.p.) route and collected splenic or placental tissues at 3, 7, or 13 days postinfection (dpi) (Fig. 1A). RNA was extracted from the tissues for assays of inflammatory gene expression via reverse transcription-quantitative PCR (qRT-PCR). Transcripts for C-X-C chemokines, a group of inflammatory mediators previously shown to be induced during B. abortus infection of bovine placentomes (13), were more highly upregulated in the mouse spleen than in the placenta at 13 dpi. These included C-X-C motif ligand 1 (*Cxcl1*) (also known as KC) and CXCL2 (*Cxcl2*) (also known as macrophage inflammatory protein 2 [MIP2]) (Fig. 1B and C). In contrast, *Ifng* and *Tnfa* were more highly upregulated in placental tissue than in the spleen at 13 dpi (Fig. 1D and E). *Ifng* has been shown previously to contribute to fetal loss in this model (4). However, Brucella spp. have been shown to evade *Tnfa* during systemic infection, so the observation that its induction in the placenta was 24-fold higher than that in the spleen was particularly striking (Fig. 1E) (4, 14–16). The *Tnfa* expression levels in the infected placenta peaked at 13 days postinfection, while the splenic *Tnfa* transcription levels in the infected spleen remained relatively low throughout the course of the experiment (Fig. 1E). The lack of *Tnfa* induction in the spleen, in which B. abortus is associated primarily with myeloid cells, was consistent with previous reports showing that Brucella spp. evade the induction of tumor necrosis factor alpha (TNF-α) production in macrophages (17, 18). These data suggested that elevated TNF-α is a specific feature of placental Brucella infection.

### *Tnfa* expression in the placenta was dependent on the induction of ER stress.

**FIG 2:**
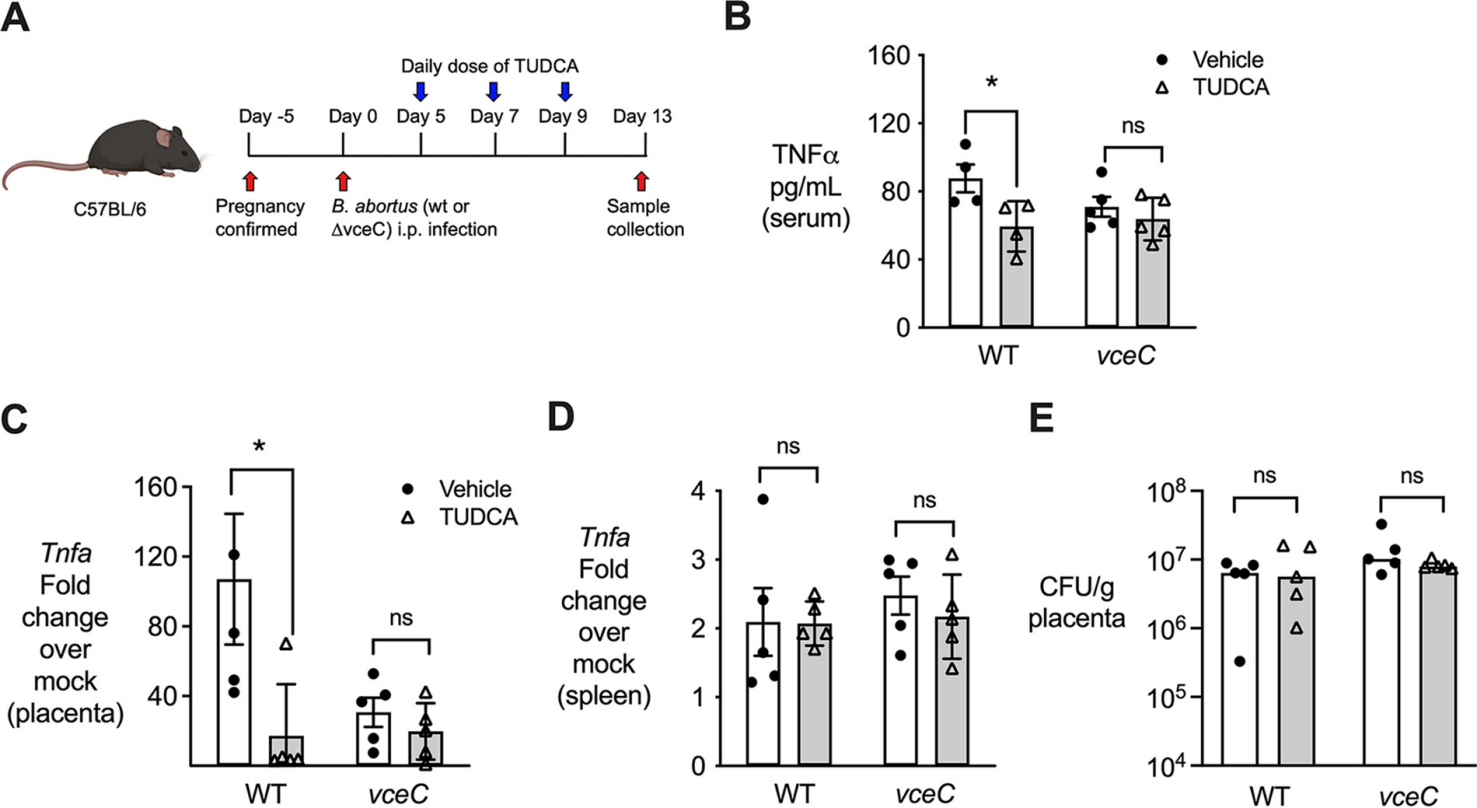
ER stress drives placental expression of *Tnfa*. (A) Schematic representation of the experiment. (B) The effect of TUDCA treatment on circulating TNF-α levels in pregnant mice infected with wild-type (WT) B. abortus or its isogenic *vceC* mutant for 13 days was determined by an ELISA. *, *P < *0.05 (using a Mann-Whitney U test). (C and D) The effect of TUDCA treatment on the transcription of *Tnfa* in placentas (C) or spleens (D) of pregnant mice was assessed by qRT-PCR (*n* = 5). Values represent means ± SEM. (E) B. abortus colonization of placentas from infected and treated mice. Differences between groups in panels C to E were compared using Student’s *t* test on log-transformed CFU data. ns, not significant.

Our previous results indicated a role for VceC, a type IV secretion system (T4SS) effector protein that induces endoplasmic reticulum (ER) stress in infected host cells, in placental inflammation and abortion caused by B. abortus in mice (9, 19). Therefore, to determine whether VceC played a role in TNF-α induction, we inoculated pregnant mice with wild-type B. abortus 2308 or an isogenic *vceC* mutant, MDJ32 (19) (Fig. 1F). The induction of *Tnfa* expression was significantly reduced in placentas infected with the *vceC* mutant compared to that in placentas of wild-type-infected mice, suggesting that VceC plays a role in eliciting *Tnfa* expression during Brucella infection (Fig. 1F). Therefore, given the link between VceC and *Tnfa* expression, we tested whether the induction of ER stress pathways by VceC was important for the induction of *Tnfa* expression. To this end, we infected pregnant mice with B. abortus and treated half the mice with the ER stress inhibitor tauroursodeoxycholic acid (TUDCA) at 5 days, 7 days, and 9 days postinfection. On day 13, tissues were collected for analysis of inflammation by qRT-PCR (Fig. 2A). TUDCA treatment reduced circulating TNF-α levels significantly in pregnant mice infected with wild-type B. abortus but had no effect on serum TNF-α levels in pregnant mice infected with the *vceC* mutant (Fig. 2B). Mice infected with wild-type B. abortus exhibited marked *Tnfa* induction in the placenta, which was largely eliminated by treatment with TUDCA (Fig. 2C). However, TUDCA treatment had no effect on the low level of placental *Tnfa* induction by the *vceC* mutant (Fig. 2C), suggesting that VceC-mediated ER stress played a role in the induction of *Tnfa* expression. Moreover, the TUDCA-mediated reduction in *Tnfa* transcripts was specific to the placenta (Fig. 2C) since no significant effect of TUDCA treatment was observed in infected spleens (Fig. 2D). Taken together, these results indicated that VceC, by eliciting ER stress, upregulated *Tnfa* expression in the B. abortus-infected placenta.

### Blockade of TNF-α prevents placental pathology.

**FIG 3:**
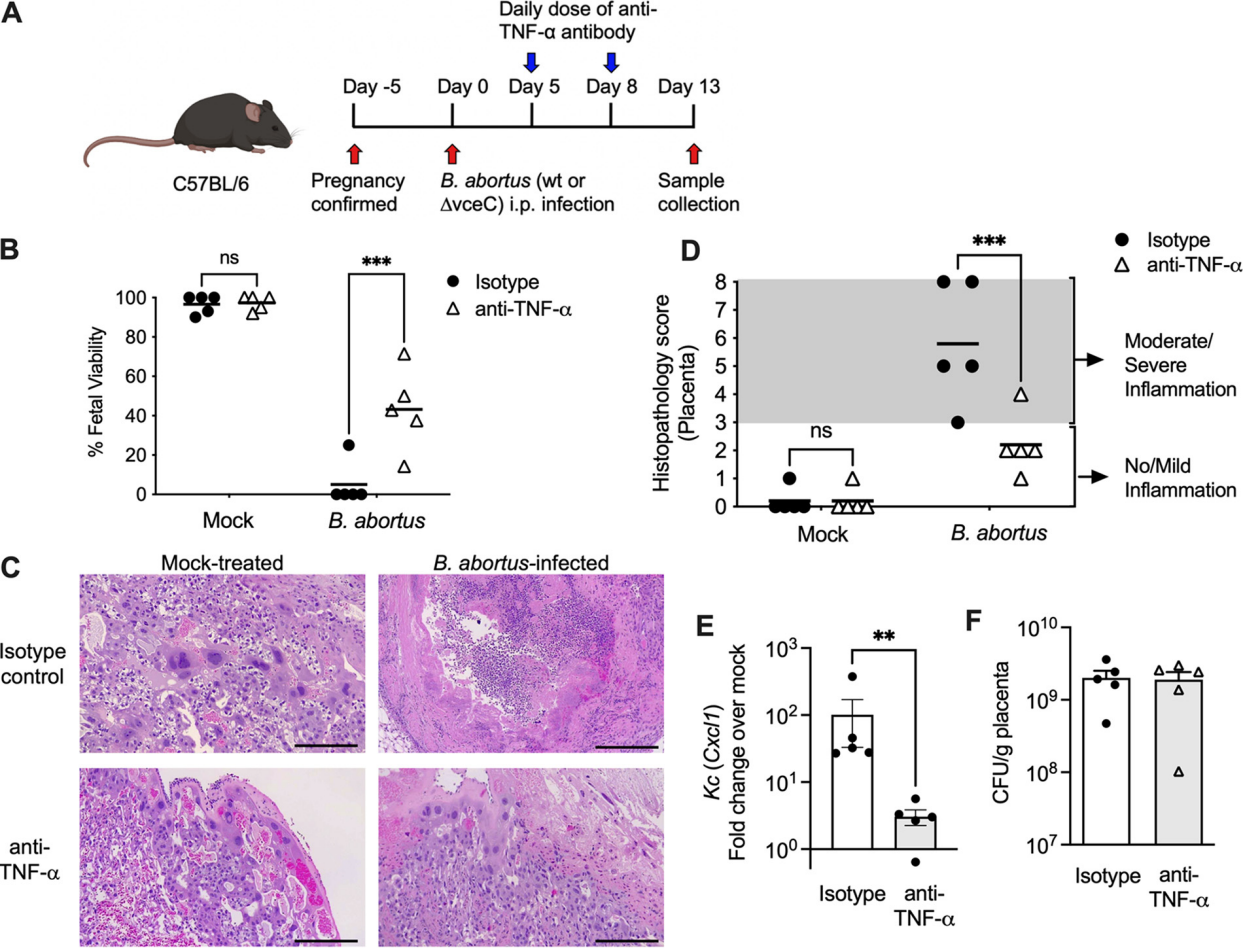
Blockade of TNF-α reduces placental pathology and increases fetal viability. (A) Schematic representation of the experiment. (B) Fetal viability of pregnant mice infected with wild-type B. abortus for 13 days and treated with anti-TNF-α antibody (0.5 mg/mouse) or an IgG isotype control (0.5 mg/mouse). Values represent data for individual dams and means (black dashes) (*n* = 5). ***, *P* < 0.0001 (using two-way analysis of variance [ANOVA] with Sidak’s multiple-comparison test). (C) Representative micrographs of placental histopathology from experimental groups showing intact trophoblasts and reduced neutrophilic inflammation in infected mice treated with anti-TNF-α. (D) Blind histopathology scoring of placental tissue. Values represent data for individual mice and means (black dashes) (*n* = 5). ***, *P* < 0.0001 (using two-way ANOVA with Sidak’s multiple-comparison test). (E) Transcriptional levels of *Kc* from placentas of pregnant mice infected with wild-type B. abortus for 13 days and treated with anti-TNF-α antibody (0.5 mg/mouse) or the IgG isotype control (0.5 mg/mouse) (*n* = 5). Values represent means ± SEM. *, *P < *0.05 (using unpaired Student’s *t* test). (F) CFU counts of B. abortus in placentas from pregnant mice infected with wild-type B. abortus for 13 days and treated with anti-TNF-α antibody (0.5 mg/mouse) or the IgG isotype control (0.5 mg/mouse). Values represent means ± SEM.

One of the major symptoms caused by B. abortus in its natural pregnant host is an acute severe inflammatory response that results in abortion and reduced fertility (2). Since *Tnfa* expression was increased during placental B. abortus infection, we investigated whether it mediates fetal loss in the pregnant mouse model. Mice infected with wild-type B. abortus for 13 days were treated with anti-TNF-α or an isotype control antibody (Fig. 3A), and the percent viability of pups was measured based on the presence of fetal movement and heartbeat and fetal size and skin color as previously described (9). While pups of dams inoculated with wild-type B. abortus were mostly deceased, anti-TNF-α treatment partially restored fetal viability (Fig. 3B). Moreover, as shown in Fig. 3C and D, placentas from wild-type B. abortus-infected mice treated by TNF-α blockade exhibited histological evidence of mild or absent placental inflammation, including an increased abundance of intact trophoblasts and a reduction of neutrophil influx, as assessed by histopathology scoring in a blind manner. Furthermore, we noted a reduction in the expression of the neutrophil chemoattractant *Kc* (*Cxcl1*) (Fig. 3E). TNF-α blockade did not affect the recovery of B. abortus from the placenta, as shown by the similar CFU recovered from the control and anti-TNF-α-treated infected placentas (Fig. 3F), suggesting that the induction of placental inflammation by TNF-α does not promote the replication of B. abortus in its placental niche. Taken together, these results indicated that TNF-α plays an important role in the severe inflammatory response triggered by B. abortus infection of the mouse placenta.

## DISCUSSION

Studies of human placental tissues have shown that TNF-α is present in the majority of normal pregnancies and peaks in abundance in the second trimester, suggesting a physiological role for this cytokine during gestation (20). However, while TNF-α production subsides in normal pregnancy by the third trimester, its production during late gestational stages has been observed under pathological conditions such as preeclampsia (21) as well as in placental infections with human cytomegalovirus (HCMV) (22). Similar to B. abortus in cattle, placental HCMV infection leads to trophoblast cell death, fetal growth restriction, and fetal pathology (23), suggesting that similar mechanisms may underlie the adverse pregnancy outcomes associated with these placental infections.

The results of this study show that B. abortus infection of pregnant mice results in the induction of *Tnfa* in the infected placenta during late gestation, where it contributes to severe inflammatory responses that result in placental pathology and fetal loss. The results also identify the T4SS effector VceC, which has previously been shown to induce placental inflammation and fetal loss in the mouse model (9), as a factor driving placental TNF-α production, via the induction of ER stress. This study advances our understanding of the links between B. abortus infection and fetal loss by identifying TNF-α as an essential host factor driving placental inflammation.

During systemic infection, human-pathogenic Brucella spp. are able to evade host surveillance mechanisms that drive the production of TNF-α by phagocytes. In Brucella suis, this property has been attributed to the outer membrane protein Omp25, which interacts with SLAMF1 on macrophages and dendritic cells to dampen TNF-α production (18, 24, 25), thereby inhibiting dendritic cell maturation and antigen presentation. Thus, during systemic infection, in which B. abortus is harbored primarily within cells of the myeloid lineage, *Tnfa* expression is only mildly elevated. However, despite the ability of Brucella spp. to inhibit its induction, TNF-α appears to contribute to the control of systemic infection since its depletion prior to infection of mice with the B. abortus vaccine strain 19 exacerbated the early stage of infection (10). It is unclear why increased TNF-α production was specific to the placenta of infected mice; however, some contributing factors may be related to the tissue context, such as the death of infected trophoblasts, which could release damage-associated molecular patterns (DAMPs) (9). Alternatively, the high bacterial loads of B. abortus in the placenta compared to those in splenic tissue or tissue-specific gene expression programs of B. abortus may drive this inflammatory response. More work will be needed to unravel these important questions about the interactions between B. abortus and its host in the placental growth niche.

Our finding of TNF-α production in the infected murine placenta is consistent with previous reports, including a study of the B. abortus vaccine strain RB51 in pregnant cattle, in which TNF-α was detected within placental trophoblasts within the inflamed placenta (7), as well as a study of canine placental explants infected with Brucella canis (14). However, bovine placental explants inoculated with B. abortus 2308 were not reported to upregulate *TNFA* during the first 4 h of infection, suggesting that either this response takes more time to develop or there may be differences between mice and cattle in this response (13). *In vitro* studies using human cell lines suggest that macrophages and neutrophils may also produce TNF-α during placental infection to drive an inflammatory response in trophoblast cells, in which B. abortus is able to replicate (15, 26). Since B. abortus resides in all of these cell types, trophoblasts, macrophages, and neutrophils, during infection (27), it is likely that reciprocal interactions between multiple cell types in the placenta drive TNF-α production, which may explain the striking reduction in placental inflammation observed after TNF-α neutralization in B. abortus-infected mice (Fig. 3).

The requirement of a B. abortus T4SS effector for the induction of TNF-α and fetal loss seems at first counterintuitive since pyogenic inflammation would be predicted to decrease bacterial colonization. However, the blockade of TNF-α, deletion of *vceC*, or suppression of ER stress had no effect on bacterial colonization, suggesting that the inflammatory response neither benefits nor is detrimental to bacterial fitness in the placenta. On the contrary, in the bovine host (in contrast to the mouse), inflammation leads to the expulsion of the fetus, which may exploit bovine social behaviors to promote transmission to the next host. Therefore, if these findings with the mouse model hold true in cattle, VceC and other B. abortus virulence factors may serve as transmission factors, eliciting a TNF-α response that drives placental inflammation and abortion.

## MATERIALS AND METHODS

### Bacterial strains, media, and culture conditions.

Bacterial strains used in this study were the virulent strain B. abortus 2308 and an isogenic mutant carrying a deletion of *vceC* (MDJ32) (28). The culture media used for B. abortus were tryptic soy agar (TSA; Difco/Becton, Dickinson, Sparks, MD), tryptic soy broth, or TSA plus 5% blood for bacterial inocula for mouse infection. Experiments with B. abortus were performed in a biosafety level 3 laboratory according to standard operating procedures reviewed and approved by Institutional Biosafety Committees, in compliance with NIH guidelines and CDC Select Agent Program regulations.

### Ethics statement.

Experiments with mice were conducted according to recommendations in the *Guide for Care and Use of Laboratory Animals* of the National Institutes of Health (29) and were approved by the Institutional Animal Care and Use Committees at the University of California at Davis under protocol number 21256.

### Animal experiments.

A murine placental infection model was utilized based on previous research (4). In short, 8- to 10-week-old female C57BL/6J mice were mated with male C57BL/6J mice, and pregnancy was confirmed by the presence of a vaginal plug. Five days after mating, the pregnant mice were then mock infected or infected i.p. with 10^5^ CFU of B. abortus 2308 or its isogenic *virB2* or *vceC* mutant. After infection, mice were kept in an animal biosafety level 3 laboratory throughout the course of the experiment. On the day of the necropsy (13 days after infection), mice were euthanized by CO_2_ asphyxiation, and the spleen and placenta were collected. Pup viability was evaluated based on the presence of fetal movement and heartbeat and fetal size and skin color as previously described (9), and the percent viability was calculated as (number of viable pups per litter/total number of pups per litter) × 100. Placental tissues were collected for bacteriology, gene expression analysis, and histopathological analysis. For treatment with the ER stress inhibitor tauroursodeoxycholate (TUDCA), mice were treated i.p. at days 5, 7, and 9 postinfection with a daily dose of 250 mg/kg of body weight of TUDCA (Sigma-Aldrich, St. Louis, MO) or the vehicle control. For TNF-α blockade experiments, mice were treated i.p. at days 5 and 8 postinfection with a daily dose of 0.5 mg/mouse of Leaf purified anti-mouse TNF-α antibody (BioLegend, San Diego, CA) or Leaf purified rat IgG1 isotype control antibody (BioLegend, San Diego, CA).

### ELISA.

TNF-α levels in the serum samples from B. abortus-infected pregnant C57BL/6J mice were measured by an indirect enzyme-linked immunosorbent assay (ELISA) (eBioscience, San Diego, CA) according to the manufacturer’s instructions.

### RT-PCR and real-time PCR analyses.

RNA from mouse tissue was isolated using Tri reagent (Molecular Research Center, Cincinnati, OH) according to the manufacturer’s instructions. cDNA was made by reverse transcription of 1 μg of DNase-treated RNA with TaqMan reverse transcription reagent (Applied Biosystems). A volume of 4 μL of cDNA was used as the template for each real-time PCR in a total reaction volume of 25 μL. Real-time PCR was performed using SYBR green and an ABI 7900 RT-PCR machine (Applied Biosystems). Primer sequences used in this study are listed in Table 1. The fold change in mRNA levels was analyzed using the comparative threshold cycle (*C_T_*) method. Target gene transcription was normalized to the levels of β-actin mRNA.

**TABLE 1:** Sequences of quantitative PCR primers used in this study

Insert	Primer	Sequence
Mouse β-actin	β-Actin_F	CCAGGGAGGAAGAGGATGCGG
	β-Actin_R	GCTGAGAGGGAAATCGTGCGTG

Mouse *Tnfa*	Tnfa_F	AGCCAGGAGGGAGAACAGAAAC
	Tnfa_R	CCAGTGAGTGAAAGGGACAGAACC

Mouse *Mip2*	mMip2_F	GCGCCCAGACAGAAGTCATAG
	mMip2_R	AGCCTTGCCTTTGTTCAGTATC

Mouse *Kc*	Kc_F	TGCACCCAAACCGAAGTCAT
	Kc_R	TTGTCAGAAGCCAGCGTTCAC

Mouse *Ifng*	Ifng_F	TCAAGTGGCATAGATGTGGAAGAA
	Ifng_R	TGGCTCTGCAGGATTTTCATG

### Histopathology.

Histopathology scoring was performed as previously described (9). In short, formalin-fixed spleen and placenta tissue sections were stained with hematoxylin and eosin, and a veterinary pathologist performed an evaluation in a blind manner using previously described criteria (27). The histopathology score was determined as cells presenting a highly basophilic pyknotic nucleus and acidophilic cytoplasm, and a score from 0 to 3 was given according to the intensity and distribution of dead cells in the tissue (0, no cell death; 1, mild focal cell death; 2, moderate, multifocal cell death; 3, severe, multifocal to diffuse cell death).
